# Ultrasound Supported Galvanostatic Deposition of Zn Coatings Reinforced with Nano-, Submicro-, and Micro-SiC Particles—Weak Acidic Chloride Baths

**DOI:** 10.3390/ma14113033

**Published:** 2021-06-02

**Authors:** Krzysztof Mech, Mateusz Marzec, Konrad Szaciłowski

**Affiliations:** Academic Centre for Materials and Nanotechnology, AGH University of Science and Technology, al. A. Mickiewicza 30, 30-059 Krakow, Poland; mmarzec@agh.edu.pl (M.M.); szacilow@agh.edu.pl (K.S.)

**Keywords:** electrodeposition, composite coatings, Zn-SiC composites, ultrasounds

## Abstract

In this paper, we present results concerning the electrochemical deposition of Zn-SiC composite coatings reinforced with nano-, submicro-, and microparticles. The influence of current density, particle size, and ultrasound on functional parameters which are especially important from a practical point of view (i.e., concentration of particles in coatings, current efficiency, morphology, reflectivity, roughness, hardness, and corrosion resistance) are investigated and discussed. Coatings were deposited from commercial, chloride-based electrolytes dedicated for the deposition of Zn coatings in a weakly acidic environment. Electrodeposited composites contained up to 1.58, 4.08, and 1.15 wt. % of SiC for coatings reinforced with nano, submicro, and micrometric particles, respectively. The process proceeded with relatively high efficiency, exceeding 80% in almost all cases. The results indicate that ultrasounds strongly increase Faradaic efficiency and affect the kinetics of electrode processes and the properties of synthesized coatings. Moreover, the obtained results show that it is possible to synthesize composite coatings with slightly higher mechanical properties while retaining corrosion resistance compared to metallic Zn coatings.

## 1. Introduction

Zinc-based materials have been in great demand for decades. Every year, there are many publications on zinc-based materials analyzed in the context of applications ranging from metallic anti-corrosion coatings [1,2,3,4,5] to protective composite coatings [1,6,7,8], decorative coatings [9,10], biodegradable materials [11,12], alloys with increased corrosion resistance [13,14], rechargeable aqueous batteries [15,16], and semiconductors [17,18,19]. Zinc coatings are most commonly used for the anti-corrosion protection of steel constructions. Zinc, due to its physical and chemical properties, location in the electrochemical series, and low cost, is an excellent alternative to other types of corrosion protection. There are two common methods of applying it. Simple construction elements, usually thick-walled, where special attention is paid to their functional properties and not aesthetic values, are coated using the hot-dip coating process, which consists of immersing the element in liquid metal and then removing the excess of zinc. In this process, intermetallic phases are formed between the surface layer of an element and the layer of crystallized zinc, and these, in combination with the surface layer of metallic zinc, create a very effective anti-corrosion coating. However, in this process, it is not possible to cover thin-walled elements which, due to the high temperature of the zinc bath (approx. 470 °C), could be deformed. Moreover, due to their poor wettability, this technique makes it impossible to apply composite coatings reinforced with ceramic particles. The electroplating process is used in applications where precision, dimensional tolerance, high corrosion resistance, abrasion and scratch resistance, high aesthetic qualities, and adequate reflectivity of coatings are required. Depending on the intended results, weakly acidic or alkaline zinc plating technologies are used commercially. The use of aqueous solutions, due to the much lower surface tension of the solvent, with the selection of appropriate parameters, allows us to obtain a colloidal electrolyte with a dispersed distribution of reinforcing particles. Hence, currently, in industrial practices aimed at obtaining composite coatings with intended functional properties, the electroplating process is mainly used.

However, it is necessary to mention the disadvantages of zinc coatings, which include susceptibility to darkening, stains, and reduced corrosion resistance in aggressive environments. Metal zinc coatings without a surface conversion layer, due to their high corrosion rate in an acidic environment, are used only for products operating in environments with a pH from 6 to 12 [20]. The phenomenon of white rust (zinc oxides) significantly reduces the life-time of such coatings. Therefore, in commonly used industrial practice, after the formation of a zinc layer, a conversion layer is produced using the chromating process. The electrochemical method is the most frequently used in the process of obtaining composite coatings with a metallic matrix and ceramic reinforcement. The addition of ceramic particles to metallic coatings has a positive effect on the improvement of the mechanical, tribological, and anti-corrosion properties of such coatings. In the literature, there are reports confirming the positive effects of the addition of Al_2_O_3_ nanoparticles or carbon nanotubes on the improvement of the corrosion resistance of Zn-Ni alloys [21,22]. The positive effect of addition of ceramic particles on increase the corrosion resistance of Zn matrices was also observed in composites reinforced with ceramic nanoparticles such as ZrO_2_, TiO_2_, or CeO_2_ [23,24,25].

In the literature, there are many works devoted to the preparation of Zn-SiC coatings reinforced with particles of various sizes using the electrochemical method. Sajjadnejad et al. noted the positive effect of the addition of ceramic nanoparticles in the Zn-SiC composite on shifting the value of the corrosion potential into the more electropositive range. For the synthesis of composite coatings, they used a pulsed current technique, and the electrolyte was mechanically mixed to ensure an homogenous distribution of ceramic particles in the electrolyte volume [26]. Al-Dhire et al. analyzed the effect of current density on the corrosion resistance and mechanical properties of Zn-SiC (2 µm) coatings deposited from sulfate baths [27]. Reventi et al. researched the preparation of Zn-SiC nanocomposites from weakly acidic chloride baths [28]. Recently, Kazimierczak et al. published several papers which indicated the possibility of synthesizing Zn-SiC coatings from citrate electrolytes [29,30,31]. The number of published papers shows that zinc electroplating is still an active research field and work is still underway on a synthesis method that obtains coatings with much higher corrosion resistance than the zinc coatings currently used.

The properties of composite coatings depend on many factors, such as current density, the composition of the electroplating bath, the concentration of ceramic particles, the presence of brightening additives, temperature, and intensity of electrolyte mixing [28]. Apart from their size, the kinetics of particle incorporation into the cathode deposit is also influenced by the types of ceramic particles involved. The increase in the rate of incorporation of particles into the metallic matrix is positively influenced by an increase in the concentration of particles in the electrolyte, a decrease in the concentration of electroactive components in the bath, a decrease in the particle size, and the conduct of the electrolysis process under pulsed current conditions [32]. Moreover, it was observed that the use of ultrasound during electrolysis reduces the agglomeration of particles, affecting the homogeneous distribution of reinforcing particles in the matrix while improving the microhardness and wear resistance of the synthesized coatings [32].

The use of ultrasound during electrolysis can prevent the agglomeration of reinforcement particles and increase the coherence of the reinforcing phase within the matrix, which is very important due to the later functional properties of the coatings. The use of ultrasound may also affect the kinetics of matrix material growth and the incorporation of ceramic particles.

The results presented in this paper are devoted to the analysis of the influence of the synthesis conditions, i.e., current density, SiC particle size, and ultrasound configuration, on the composition, electrolysis efficiency, morphology, and various parameters important from the application point of view, such as the roughness, reflectivity, hardness, and corrosion resistance of the coatings. It is noteworthy that the tests were carried out with the use of a commercially used electrolyte intended for the application of zinc coatings in a weak-acid environment, modified with the addition of SiC particles.

## 2. Materials and Methods

Coatings were deposited from an AZUR HT5 (KIESOW DR. BRINKMANN GmbH & Co. KG, Detmold, Germany) commercial bath dedicated to electrodeposition of anticorrosive protective zinc coatings. For deposition of composite coatings, the bath was modified with the addition of SiC nanoparticles (SiC, 99+ %, 45–65 nm, cubic), submicrometer particles (SiC, beta, sub-micron powder, 99+ %, D < 1 μm/APS 800 nm), and SiC microparticles (99+ %, 1–40 μm, US Research Nanomaterials, Inc., Houston, TX, USA), respectively.

The base AZUR HT5 electrolyte was prepared by the dissolution of 30 g/L ZnCl_2_ (reagent grade, KrakChemia, Kraków, Poland), 145 g/L of KCl (pure for analysis, POCH), an 25 g/L H_3_BO_3_ (pure for analysis, POCH) in 700 mL of demineralized water at 60 °C. Finally, the electrolyte was supplemented with the Azur HT 5 Bright—brightness controlling (0.5 mL/L)—and base—containing wetting agents and basic brighteners (40 mL/L)—additives. Then the volume of electrolyte was filled up to 1 L. After cooling the solution down to 30 °C, the pH was adjusted to 5.5 with the addition of 15 HCl or 10% KOH solutions.

Coatings were deposited at galvanostatic conditions onto the surface of a low-carbon steel sheet of DC01A type ((1.0330) EN 10130/10131) with a surface area of 3.14 cm^2^.

A zinc sheet 16 cm^2^ was applied as the soluble anode. The distance between the anode and cathode was 7 cm. The sonotrode (VS 70 T, Bandelin Sonoplus HD3200, Berlin, Germany) was placed from the top at an equal distance from the cathode and the anode. The electrolyte was recirculated and cooled to room temperature in a 250 mL reservoir to eliminate the electrolyte heating effect. The cooling system was composed of an IKA HRC 2 Control thermostat and a vessel with a water jacket.

Before electrodeposition, the cathode surface was prepared as follows: the steel sheet was chemically degreased in Surfclean 900 for 10 min at 70 °C, then rinsed with demineralized water at 50 °C. In the next step, the cathode surface was degreased electrochemically in Ekasit E57 electrolyte at 70 °C, and at 5 A/dm^2^ for 5 min and rinsed with demineralized water at 50 °C. The last treatment step before electrolysis was a chemical etching of the steel sheet surface in 10% HCl at 25 °C for 5 min. After etching, the cathode surface was rinsed with demineralized water once again. The steel surface prepared in this way was electrochemically covered with Zn or Zn-SiC coatings containing SiC particles of various sizes.

The Zn and Zn-SiC protective layers were deposited with and without the presence of ultrasound at a current density from 1 to 3 A/dm^2^ with a step of 1 A/dm^2^. The temperature of the electrolyte was kept at 30 °C using the IKA HRC 2 Control thermostat (IKA, Staufen, Germany). Colloid electrolytes contained 5 g/L of SiC particles of particular fractions.

The electrokinetic potential (ξ) of the SiC particles was measured using a Malvern Zetasizer, a NANO ZS (Malvern Instruments Ltd., Malvern, UK) apparatus equipped with a He-Ne (λ = 633 nm) laser. The ξ was determined based on 3 measurements. Before measurement, colloid electrolytes were dispersed in the presence of ultrasounds for 1 h. Measurements were performed at room temperature and stabilized for 120 s. The ξ values were calculated using the Smoluchowski equation.

Elemental analysis was performed using the wavelength dispersive X-ray fluorescence spectrometer (WD-XRF) Rigaku ZSX Primus IV (Rigaku, Tokyo, Japan). The system uses a 4 kW rhodium tube equipped with a window of 30-micron thickness, a maximum accelerating voltage of 60 kV, and a maximum current of 150 mA. Samples during analyses were rotated at 30 rpm. Semi-quantitative analysis (SQX) was performed using ZSX Software.

A Quanta 3D 200i scanning electron microscope (FEI, Hillsboro, OR, USA) was used for the characterization of the morphology of the electrodeposited composite coatings. Observations were performed at a magnification of 2.5k.

Analysis of coatings reflectivity was performed based on the diffusion reflectance spectra recorded using a PerkinElmer UV-Vis-NIR Lambda 750 spectrometer equipped with an integrating sphere (100 mm InGaAs) (Perkinelmer, Waltham, MA, USA). Spectra were recorded in the range of 195–2250 nm versus the Spectralon^®^ reference sample.

The roughness analysis of the samples was carried out using a Bruker DekTakXT profilometer with Vision 64 software (Bruker, Billerica, MA, USA). The Ra parameter (arithmetic mean deviation of the profile (Z_i_) from the mean line) was determined based on the EN ISO 4287 standard. Measurements were carried out in contact mode. Ra values were determined based on 10 recorded linear profiles.

The hardness measurements were carried out with the use of Tukon 2500 hardness tester (Wilson Hardness, Lake Bluff, IL, USA) using the Vickers method. Measurements were carried out with a low load force.

Corrosion tests were performed with the use of a BioLogic SP-200 potentiostat/galvanostat in a standard three-electrode system. Analysis of corrosion properties as based on open circuit potential (*OCP*) and the potentiodynamic curves. The potentiodynamic curves were recorded starting from −200 mV to +200 mV vs. *OCP*, with a sweep rate of 2 mV/s. Tests were performed in 0.5 M NaCl. Leakless Ag/AgCl electrode was applied as a reference electrode (0.214 V vs. NHE) and a Pt gauze electrode was applied as a counter electrode. Corrosion potential (*E_corr_*) and corrosion current (*I_corr_*) were determined using EC-Lab v.11.20 software. All electrochemical tests and electrodeposition were performed at room temperature.

Cathodic current efficiency and coating deposition rates were determined based on electrode mass changes.

## 3. Results and Discussion

The research began with an impact analysis determining the value of the electrokinetic potential of the particles in the electrolyte used. The determined values of the electrokinetic potential were 9.07 ± 1.11 mV for the SiC_nano_ particles, 6.54 ± 0.37 mV for the SiC_submicro_ particles, and 8.64 ± 1.23 mV for the SiC_micro_ particles. Regardless of the analyzed system, the determined values of the electrokinetic potential indicate their high susceptibility to agglomeration, although surfactants were added to the electrolyte.

**Figure 1:**
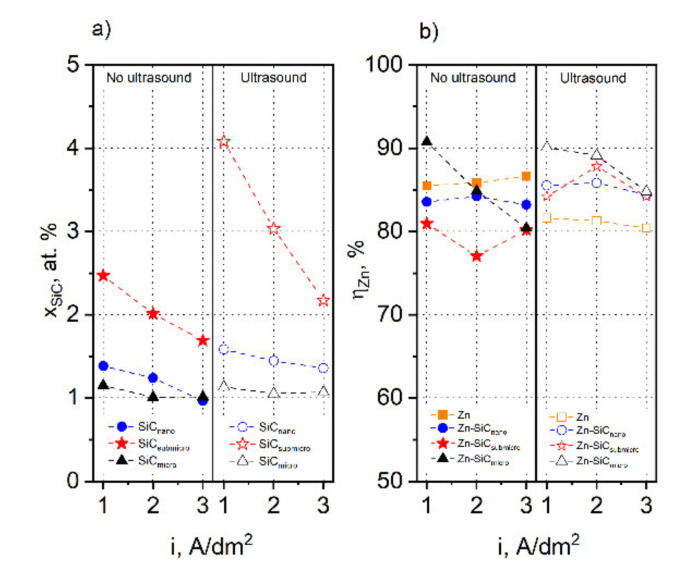
Parameters of coatings deposited at different current densities from an SiC free electrolyte and an electrolyte containing 5 g/L of SiC particles of different sizes. Coatings were deposited without and in presence of ultrasound. (**a**) SiC content in composite coatings, (**b**) cathodic current efficiency.

Figure 1a shows the results of the analysis of the chemical compositions of the coatings obtained at galvanostatic conditions for different current densities in the presence, or absence, of ultrasound. The coatings were deposited from electrolytes containing nano-, submicro-, and micrometric SiC particles. In each case, the increase in the cathode current density resulted in a decrease in the content of the ereinforcing particles in the coatings. The decrease caused by the increase in current density was most noticeable in the case of coatings deposited from an electrolyte containing submicrometric particles. It was also observed that the coatings deposited from this electrolyte exhibited the highest content of SiC particles. The coatings deposited without the presence of ultrasounds from electrolytes containing SiC_nano_ and SiC_micro_ contained very similar particle amounts. For current densities of 1 and 2 A/dm^2^, these differences amounted to approx. 0.25% at. whereas for 3 A/dm^2^ the particle content was very close.

It is known that an increase of the cathodic current accelerating the kinetics of zinc deposition and promotes the course of the hydrogen evolution reaction. It happens even despite the relatively high overpotential of zinc for HER. The kinetics of SiC particle co-deposition are strongly related to the concentration of particles in the electrolyte. Thus, at constant current conditions, where the rate of electrochemical processes increases with current density and the rate of SiC co-deposition is limited by its concentration in the electrolyte, a final effect may be noticed as a decrease of SiC content in the coatings, as was demonstrated by obtained results. The analysis of the chemical composition of the coatings deposited in the presence of ultrasounds revealed that the kinetics of SiC_submicro_ particle incorporation into the cathodic deposit was much higher than that of particles of other sizes. The same effect was observed in the case of coatings deposited without ultrasound. The highest content of SiC particles, approx. 4.1% at., was observed for the coating obtained from the electrolyte containing SiC_submicro_ particles at a current density of 1 A/dm^2^. A similar effect of the faster incorporation of submicrometric than nanometric particles was observed by Simunkowa et al. in the case of co-deposition of nickel with ZrO_2_ particles from a Watts bath [33]. The lowest concentration of micrometric particles in the coating resulted from their fast sedimentation, which was noticed during the electrodeposition process. It was observed that the application of ultrasound had a significant impact on the incorporation kinetics of nano- and micrometric particles, while the impact on the incorporation kinetics of the micrometric particles was not as noticeable. The effect of current density on the SiC_micro_ content in the coatings also was not significant, which was inferred from the very subtle differences in the contents of the particles. In the case of the electrodeposition carried out in the presence of ultrasound, the greatest effect of the current density on changes to the contents of the particles in the coatings was observed in the case of electrolytes containing SiC_submicro_ particles. The positive effect of ultrasound on the increase of the SiC_nano_ and SiC_submicro_ contents in the coatings can be associated with the reduction of their agglomeration and sedimentation, which in turn could have facilitated the transport of these particles to the electrode surface. Studies on the electrodeposition of Zn-SiC (60 nm) coatings from acidic chloride electrolytes were also performed by Reventi et al. [1]. The coatings deposited at 45 °C from the baths containing from 10 to 20 g/L of SiC contained 2.74 to 3.38 at % of SiC [28]. Kazimierczak et al. reported results for the electrodeposition of Zn-SiC coatings from citrate electrolytes containing 60 g/L of SiC particles of average particle sizes between 50 and 90 nm [29]. The coatings were deposited in a wide range of current densities from 0.5 to 5 A/dm^2^. Previous studies reported results similar to the results reported in the present work, indicated a strong influence of particle size and current density on coating composition. 

In the next stage, we examined the influence of several electrolysis parameters on the current efficiency of the electrolysis process related to the Zn matrix (Figure 1b). The efficiency of the electrolysis conducted with the use of a SiC-free electrolyte without ultrasound slightly increased with the increase of the current density, and reached values slightly higher than 85%. Except for the coating deposited at 1 A/dm^2^ from an electrolyte containing SiC_micro_ particles, the efficiency of the composite coatings was lower compared to electrodeposition of metallic zinc coatings.

Decreased current efficiency results from a concurrent hydrogen-evolution reaction. The increase in current efficiency we noted may be have related to a different mechanism of control of the electrode processes. Due to the high concentration of Zn^2+^ ions in the electrolyte and the course of reaction leading to its reduction in the activation control range and high overpotential of zinc for HER, an increase in current density accelerated the kinetics of the electrode processes related to zinc deposition and simultaneously limited kinetics of concurrent HER.

The same effect was reported for the electrodeposition of a Ni-based coating reinforced with a SiC particles, and was explained by the ability of the particles to adsorb protons, which in turn may result in the enhancement of HER. [34,35]

The presence of ultrasound caused the opposite effect, which manifested with a slight decrease in the Zn deposition efficiency with increasing current density. In the case of the electrolysis carried out under ultrasonic agitation, we observed a decrease in efficiency by approx. 4% for 1 A/dm^2^, and these differences became increasingly significant with the increase of the current density. It was reported that ultrasound efficiently removed hydrogen bubbles—one of the steps limiting the kinetics of HER—making the overall process faster [36]. This, in turn, may be responsible for the lower efficiency of metallic zinc electrodeposition in presence of ultrasound compared to a deposition without ultrasound. For the electrodeposition of composite coatings in the presence of ultrasound, the observed effect is different. The efficiency of electrolysis carried out with the use of an electrolyte containing SiC_nano_ particles remained at a similar level and amounted to approx. 83.5%. In this case, the use of ultrasound increased the electrolysis efficiency from 0.8% (3 A/dm^2^) to almost 2% (1 A/dm^2^) compared to electrodeposition conducted without ultrasound.

In the case of electrolysis carried out without ultrasound with the use of an electrolyte containing submicrometric particles, we observed lower current efficiencies than in the case of a SiC-free electrolyte. The current efficiencies in this case ranged from 77.1 for 2 A/dm^2^ to 80.84% for 1 A/dm^2^. The use of ultrasound, in this case, resulted in a significant increase in current efficiency, regardless of the current density applied, with the largest increase of 17.6% being observed for the coating deposited at a current density of 2 A/dm^2^.

The highest current efficiency of 90.7% was observed in the case of the synthesis carried out without the presence of ultrasound, at a current density of 1 A/dm^2^ from electrolyte containing SiC_micro_ particles. The efficiency of the electrolysis conducted with the use of this electrolyte decreased with the increase of the current density and reached a value of 80.3% for 3 A/dm^2^. The presence of ultrasound did not cause significant changes to the current efficiency for the electrolysis carried out at 1 A/dm^2^, while the changes observed for the other current densities amounted to as much as 4.6% for 3 A/dm^2^. It was observed that the presence of ultrasound, regardless of the particle sizes, had a significant impact on the current efficiency of the electrolysis. The current efficiency may have been the effect of the positive influence of ultrasound on the kinetics of gas desorption from the electrode surface and the kinetics of reagent transport to the electrode surface. However, current efficiency depends on many other factors. Apart from ultrasound, the number and size of particles present on the electrode surface have a significant influence on the kinetics of hydrogen evolution. The presence of ceramic particles at the electrode surface may limit the electrochemically active surface area and promote an increase in the number of centres where privileged hydrogen evolution could take place. The use of ultrasound, in turn, made the efficiency of the composite coating synthesis higher than the efficiency of zinc coating synthesis, regardless of the particle sizes and the current density applied. Additionally, in the case of coatings deposited at current densities of 2 and 3 A/dm^2^, it was observed that the efficiency of the process increased with the size of the particles present in the electrolyte. This proves the noticeable influence of ultrasonic agitation on the kinetics of the processes occurring at the electrode surface.

**Figure 2:**
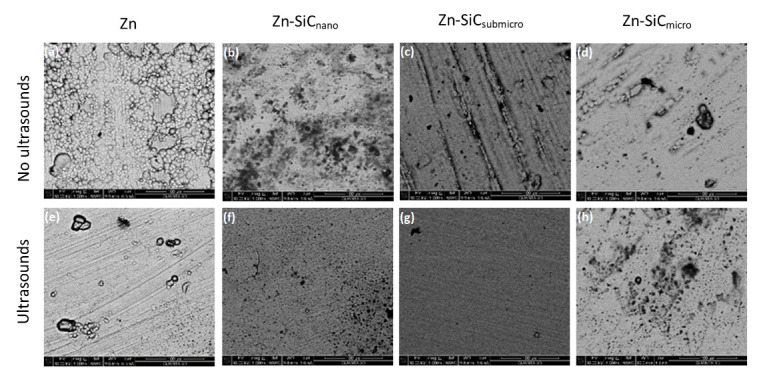
SEM images of Zn and Zn-SiC coatings deposited at 1 A/dm^2^ without (**a**–**d**) and in the presence of ultrasound (**e**–**h**) (mag. 2.5k).

Figure 2 presents the morphology of metallic Zn coatings and composite Zn-SiC coatings reinforced with particles of different sizes obtained with and without the presence of ultrasound. Due to the high content of ceramic particles, the coatings deposited at 1 A/dm^2^ were observed closely. The morphology of the coatings was similar regardless of the applied current density. On the surface of the synthesized coatings, longitudinal traces resulting from the displacement of bubbles of hydrogen may be observed. Single circular pores resulting from localized hydrogen evolution were also visible on the surface of the coatings. Based on the presented results, it can be concluded that the surface roughness of the coatings obtained in the presence of ultrasound slightly decreased. A positive effect of ultrasonic agitation on surface roughness reduction was also observed by Sheng et al. for electrodeposited Ni-Co coatings [37]. The presence of ultrasound during electrodeposition enhances mass transfer, influencing the kinetics of the electrode reactions, reducing grain size and the influencing processes of the desorption of gas bubbles from the electrode surface [38,39]. This, in turn, resulted in the reduction of the number of pores and the longitudinal traces resulting from the displacement of hydrogen bubbles, as indicated by the presented results. The positive influence of ultrasonic agitation on the morphology of nickel coatings was reported by Ginberg et al, who observed that the application of ultrasounds, depending on the applied current density, may lead to the formation of a fine-grain structure, especially at relatively high current densities [40]. 

It should be also noticed that the morphology of the deposited coatings is very different from those reported by Kazimierczak et al. (citrate bath [29]), Sajjadnejad et al. (sulfate bath [26]), and Roventi et al. (chloride baths [28]) indicating also the strong influence of the type of electrolyte on coatings morphology.

A comparative analysis of the reflectivity of zinc and composite coatings was performed based on the recorded DRS spectra. They allowed for the determination of the amount of light absorbed by the coatings in the spectral range of 380–750 nm. Based on the recorded DRS spectra obtained for the coatings, Kubelka–Munk relationships: *F*(*R*) = *k*/*s* (*s* = 2*R*—dissipation factor, *k* = (1 − *R*)^2^—molar absorption coefficient, *R*—reflectance) were plotted. The analysis of the reflective properties was carried out based on the parameter $A=\int_{1.65eV}^{3.27eV}F\left(R\right)dE$, which characterizes quantitatively the absorption of radiation in the visible range. While numerous factors influence the diffused reflectivity of surfaces (plasmonic features of metallic surfaces, roughness and other geometrical factors, chemical composition, bandgap of semiconducting nanoparticles), these data may be used to describe quantitatively the aesthetic features of the coating, which are important in various consumer applications. Therefore the Kubelka–Munk function, integrated over the whole visible range, was used as a generalized measure to evaluate the aesthetic quality of the samples.

**Figure 3:**
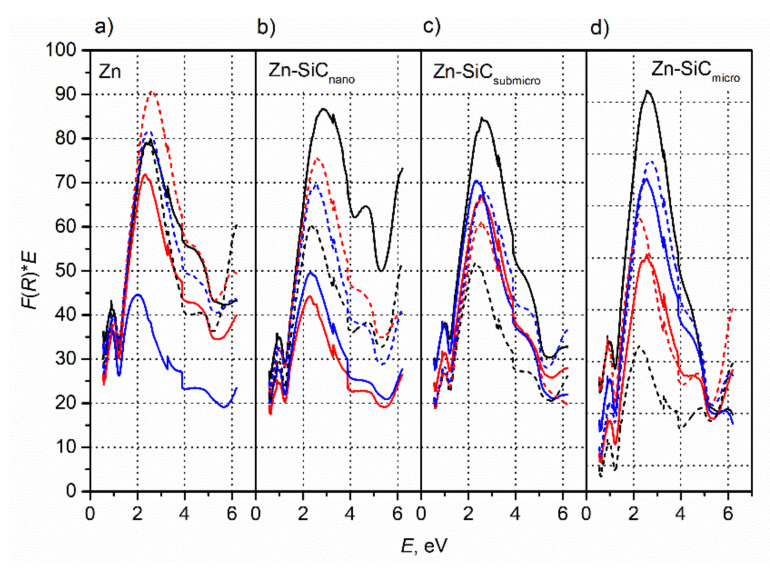
[*F*(*R*)*E] vs. *E* dependences based on DRS spectra recorded for various coatings deposited under different conditions: (**a**) Zn, (**b**) Zn-SiC_nano_, (**c**) Zn-SiC_submicro_, and (**d**) Zn-SiC_micro_ (black—1A/dm^2^, red—2 A/dm^2^, blue—3 A/dm^2^; solid lines—no ultrasound, dashed—ultrasound).

Figure 3 presents the Kubelka–Munk (F–K) relationships plotted for the Zn and Zn-SiC coatings synthesized in the presence and absence of ultrasound. Based on these relationships, the A parameter was determined for particular coatings. This parameter made it possible to analyze the influence of particle size and current density on the reflectivity of the synthesized coatings. The determined parameter values for all obtained coatings are given in Table 1. In the case of Zn coatings deposited without the presence of ultrasound, the increase in the current density caused a decrease in the A_Zn_ parameter value, indicating at the same time the lower ability of the coatings to absorb light in the visible range, thus indicating an increase in their total reflectivity. In the presence of ultrasound, the lowest A_Zn_ parameter value was observed for coatings deposited at *i* = 1 A/dm^2^. In the case of the A_Zn-SiC_nano__, A_Zn-SiC_submicro,__ and A_Zn-SiC_micro__ coatings synthesized at the presence of ultrasound, the highest reflectance was observed for *i* = 1 A/dm^2^, while for coatings deposited without ultrasound it was 2 A/dm^2^.

All composite coatings obtained without ultrasound at 3 A/dm^2^ exhibited lower reflectivity than the metallic Zn coatings. An application of ultrasound, regardless of the size of the reinforcing particles, caused significant changes in the reflectivity of coatings obtained under the same current conditions. All Zn-SiC_micro_ coatings obtained in the presence of ultrasound exhibited higher reflectivity than the metallic zinc coatings.

It may be observed that the application of ultrasound exerted the most significant effect in the increase in the reflectivity of coatings deposited at 1 A/dm^2^. At this current density, the kinetics of the electrode reactions are relatively low; applying higher current densities may increase the kinetics of concurrent processes like the reduction of hydrogen ions or even the reduction of water molecules because of a weakly acidic environment. The reduction of water molecules due to local alkalisation of near the electrode-surface area may, in turn, result in changes to the thermodynamic equilibrium, promoting the formation of oxides and hydroxides. As mentioned earlier, the kinetics of the concurrent reactions is also related to the size and concentration of SiC particles in the electrolyte. Many factors influencing surface roughness, number of pores, and compounds present on the coating surfaces make an analysis of the influence of individual electrolysis parameters on reflectivity very difficult. In the literature, there is much information concerning the positive effect of ultrasonic agitation on coatings brightness. It may be found that in the case of copper and nickel coatings, the application of ultrasound allows for the deposition of bright coatings at current densities 4-times higher than typical conditions [41]. The positive effect of ultrasonic agitation enabling deposition of bright coatings at much higher current densities was also observed by Roll et al. [42]. Previous to findings presented in this work, there was no information concerning the influence of ultrasound on the reflectivity of zinc or Zn-SiC coatings in the literature.

**Figure 4:**
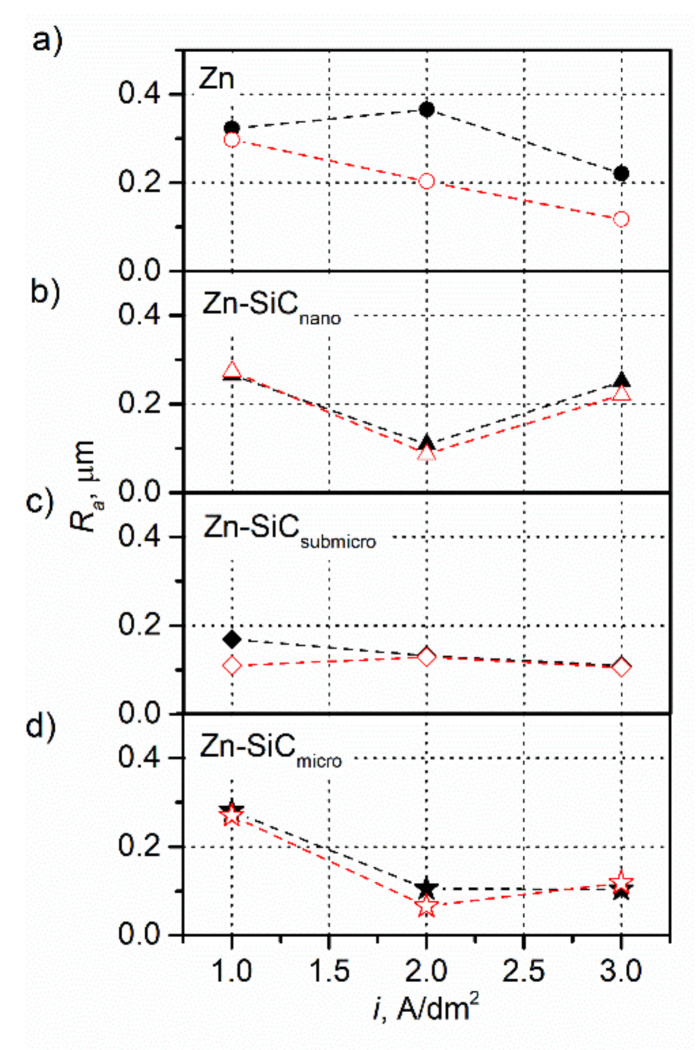
Surface roughness of deposited coatings: (**a**) Zn, (**b**) Zn-SiC_nano_, (**c**) Zn-SiC_submicro_, and (**d**) Zn-SiC_micro_ (black—no ultrasound, red—ultrasound).

The results of the profilometric measurements presented in Figure 4 show that the obtained coatings are characterized by very low surface roughness, regardless of synthesis conditions. The differences in the roughnesses of coatings containing individual particle fractions, or of the same coatings synthesized under different current conditions or ultrasound configurations are very subtle. A positive effect of the increase of the current density on the decrease in the surface roughness of metallic coatings, especially those applied in the presence of ultrasound, was observed—an increase in the current density from 1 to 3 A/dm^2^ resulted in an almost twofold decrease in the surface roughness. The differences resulting from the presence of ultrasound were the most visible for the lowest value of cathode current: *i* = 1 A/dm^2^. The roughness of the Zn-SiC_nano_ and Zn-SiC_micro_ coatings obtained at 1 A/dm^2^ were similar to the roughness of metallic Zn coatings obtained at the same current density. The roughness of all composite coatings obtained at 2 A/dm^2^, regardless of the configuration of the ultrasound, remained at a similar level, much lower than that observed for the metallic coatings. The profilometric analysis and measurements of reflectivity, which showed that the obtained coatings differed significantly in reflectivity despite their similar surface roughness, allow us to conclude that apart from surface roughness, the surface composition of the coatings, both elemental and phase, has a significant impact on reflectivity. Decreased roughness in the presence of reinforcing particles may be a consequence of a higher nucleation rate in the presence of SiC particles. Another explanation of the observed effect, proposed by Walker and Walker, may be the collapse of hydrogen bubbles and the acoustic flow at the electrode surface which, in turn, due to ultrasonic agitation, decreased the porosity and positively affected the hardness, surface homogeneity, and reflectivity of coatings [40].

**Figure 5:**
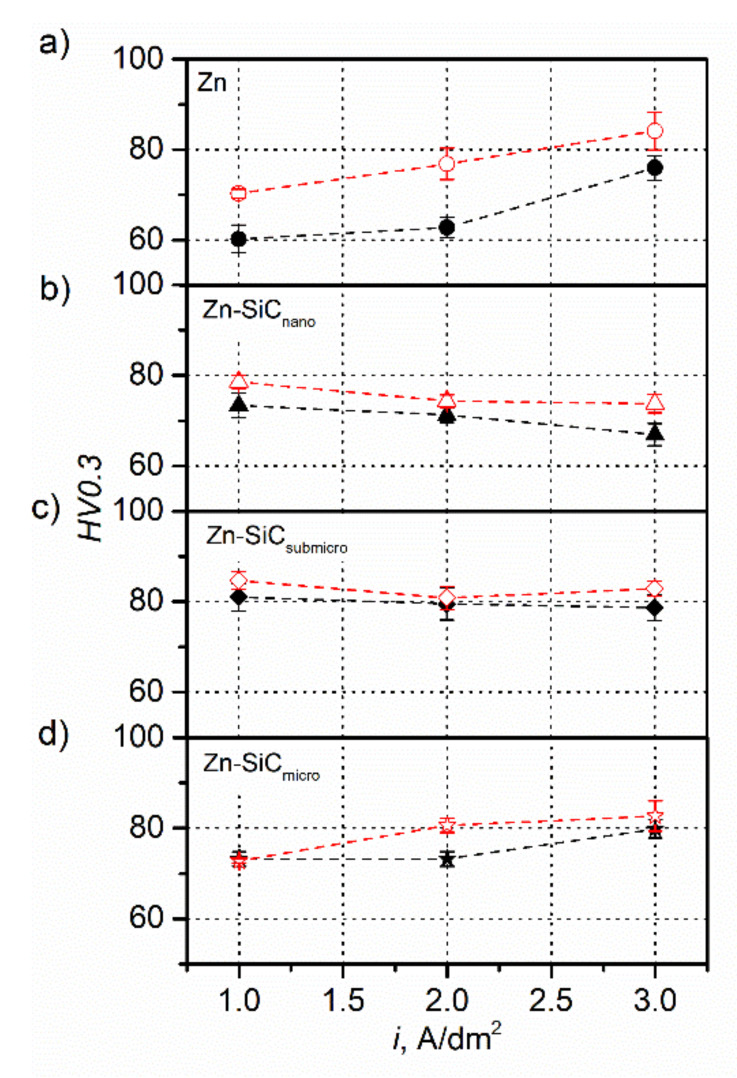
Hardness (HV0.3) of deposited coatings: (**a**) Zn, (**b**) Zn-SiC_nano_, (**c**) Zn-SiC_submicro_, and (**d**) Zn-SiC_micro_ (black—no ultrasound, red—ultrasound).

The results of the hardness measurements of the obtained coatings are shown in Figure 5. In the case of metallic coatings and composite Zn-SiC_micro_ coatings, a positive effect of the increase in the current density was observed, resulting in an increase in hardness by nearly 15 *HV0.3* in the case of the Zn coatings and by 10 *HV0.3* in the case of the Zn-SiC_micro_ coatings. This effect was visible in both the coatings deposited with and without the presence of ultrasound. In the case of the Zn-SiC_nano_ coatings, a decrease in the hardness of the coatings was observed along with an increase in the current density, while in the case of the Zn-SiC_submicro_ coatings, the *HV0.3* values remained at a similar level. The effect of ultrasonic agitation on the mechanical properties of the coatings was most visible in the case of the metallic coatings. The highest increase in the hardness of the coatings was observed for the current density of 2 A/dm^2^, by as much as about 13 *HV0.3*. A significant increase in hardness was also observed for the same current density in the case of the Zn-SiC_micro_ coatings. In the case of the coatings reinforced with nanometric and submicrometric particles, the changes were not significant. The low value of the error bars based on 10 measurements indicate the high homogeneity of the coatings. Changes in micro-hardness resulting from an increase in the current density and the presence of ultrasound may have resulted from the presence of presence of reinforcing particles and changes in the sizes of the crystallites resulting from different crystallization and growth conditions. The increase in the current density positively influenced the increase of the number of nuclei, which may have had a positive effect on the improvement of the mechanical properties of the coatings [43]. The effect that resulted from the application of ultrasonic agitation in the case of zinc coatings was previously reported in the work of Walker and Walker [40]. Pavlatou et al. explained the increasing of micro-hardness of Ni coatings reinforced with SiC particles by a reduction of the coatings grain size, modification of texture through the growth of the coatings in a preferred orientation, and by the strengthening effect coming from the presence of the reinforcing particles [44]. Muller and Kuss also postulated that ultrasound caused an increase in the number of reinforcing particles which in turn blocked the movement of dislocations and improved coatings hardness [45]. Another explanation for the increase of the micro-hardness of coatings was proposed by Walker and Walker in a work published later. They postulated that ultrasound caused deformation on the surface of growing deposits and their hardening by the impact of the shock waves coming from the implosion of cavities [46].

**Figure 6:**
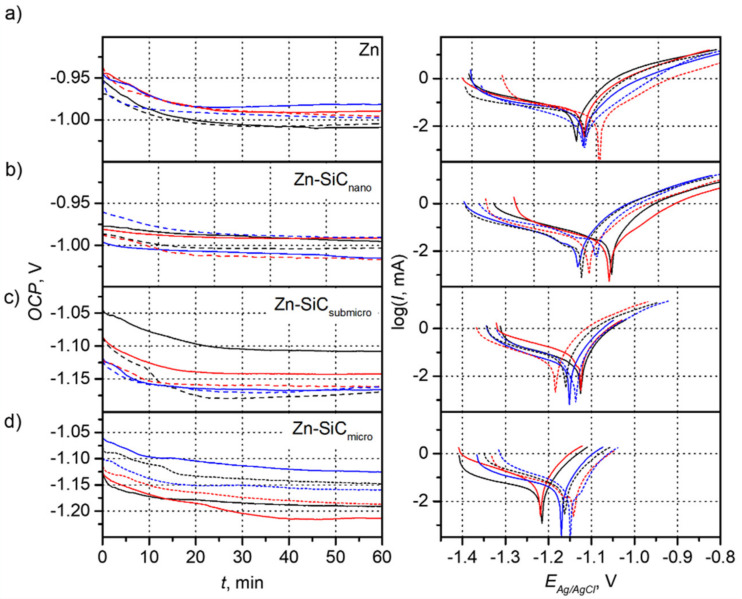
Chronopotentiometric and Tafel curves recorded for Zn and Zn-SiC coatings deposited at different conditions: (**a**) Zn, (**b**) Zn-SiC_nano_, (**c**) Zn-SiC_submicro_, and (**d**) Zn-SiC_micro_ (black—1 A/dm^2^, red—2 A/dm^2^, blue—3 A/dm^2^; solid lines—no ultrasound, dashed lines—ultrasound).

Figure 6 presents the chronopotentiometric and Tafel curves recorded for coatings obtained with and without ultrasonic agitation. The *OCP* values were determined based on the chronopotentiometric curves, while the values of *E_corr_* and *I_corr_* were determined based on the potentiodynamic curves. The obtained results are summarized in Table 2. The determined values allowed for a comparative analysis of the corrosion properties of the composite and metallic coatings. In the case of the Zn coatings, the *OCP* values, regardless of the current density used and the configuration of the ultrasound, remained almost at the same level—1.0 V. Noticeable slight differences in the value of the *OCP* potential were within the measurement error. The *E_corr_* values also remained at a similar level and indicate the similar corrosion properties of the obtained coatings. Only the differences in the values of the current intensity indicate the higher corrosion resistance of the coatings obtained in the presence of ultrasound. The lowest value of corrosion current was observed for the coating applied at 2 A/dm^2^. Almost the same *OCP* and *E_corr_* values were observed in the case of the Zn-SiC_nano_ coatings, which indicates the similar corrosion resistance of the coatings. The lowest observed values of the corrosion current remained at a similar level of approx. 17 µA and were observed in the case of coatings obtained without ultrasound at current densities of 1 and 2 A/dm^2^ and with ultrasound at a current density of 1 A/dm^2^. The Zn-SiC_submicro_ coatings were characterized by significantly higher *OCP* and *E_corr_* values by as much as about 0.1 V. The much lower corrosion resistance is also indicated by much higher values for the corrosion current. The lowest value of 31.6 µA was observed for the coating prepared at 1 A/dm^2^ without the presence of ultrasound. The Zn-SiC_micro_ coatings were characterized by *E_corr_*, *I_corr_*, and *OCP* parameters similar to those of the Zn-SiC_submicro_ coatings. The obtained results suggest that the increase in SiC particle size negatively influenced the corrosion resistance of the coatings. Sajjadnejad et al. investigated the deposition Zn-SiC coatings from sulfate electrolytes containing between 5 and 15 g/L of SiC (50 nm). The *E_corr_* values reported for coatings deposited at 5 g/L of SiC were very close to those reported in the present work [1]. Al-Dhire et al. reported that the improvement of corrosion resistance of the composite coatings may be associated with the formation of micro-galvanic cells resulting from incorporation of SiC particles into the zinc matrix. In these cells, SiC particles act as cathodes, while anodic processes take place at the surface of the zinc matrix [27]. In the reported results, the clear effect of the presence of SiC particles on corrosion resistance improvement was not observed. It should be underlined that the corrosion parameters determined resulted from many overlapping effects, including SiC presence and the current density and ultrasound effects on surface morphology, formation of zinc compounds, porosity, grain size, and internal stresses.

## 4. Conclusions

The obtained results indicated that it is possible to synthesize Zn-SiC composite coatings from weak-acid electrolytes, and confirmed that the presence of ultrasound during synthesis has a significant impact on the properties of the obtained coatings. The highest content of particles in the coatings was noted for the coatings synthesized in the presence of ultrasound from an electrolyte containing submicrometric particles at a current density of 1 A/dm^2^. It was also observed that, regardless of the size of the reinforcing particles and the ultrasound configuration, an increase in the current density caused a decrease in the content of ceramic particles in the coatings. Analysis of the reflectivity of the coatings showed that some composite coatings exhibited reflectivity similar to, or even slightly better than, that of zinc coatings. Analysis of the surface roughness showed that the obtained coatings, both metallic and composite, were characterized by a low Ra parameter. Regardless of the electrolysis conditions, the presence of ultrasound caused an improvement in the mechanical properties of the coatings. The corrosion properties of Zn-SiC_nano_ coatings were very similar to the properties of zinc coatings. The obtained results demonstrate that under certain, appropriately selected conditions, it is possible to synthesize composite coatings with corrosion resistance similar to that of Zn coatings and, with better mechanical properties, and of higher aesthetic value.

## Figures and Tables

**Table 1:** A factor values determined based on [*F*(*R*)*E](*E*) dependences (Figure 3) for Zn and Zn-SiC coatings deposited from weak acid electrolytes.

*i*, A/dm^2^	Ultrasound	A_Zn_	A_Zn-SiC_nano__	A_Zn-SiC_submicro__	A_Zn-SiC_micro__
1	No	116.6	122.5	122.1	133.9
2		103.4	62.8	96.6	87.7
3		61.4	71.3	100.9	109.1
1	Yes	112.9	86.7	73.6	61.4
2		130.6	108.2	87.6	95.4
3		118.4	99.9	97.2	112.2

**Table 2:** Values of OCP, E_corr_, and I_corr_ determined for particular coatings based on data presented in Figure 6.

		Zn			Zn-SiC_nano_			Zn-SiC_submicro_			Zn-SiC_micro_		
*i*, A/dm^2^	Ultrasound	*OCP*, V	*E_corr_*, V	*I_corr_*, µA	*OCP*, V	*E_corr_*, V	*I_corr_*, µA	*OCP*, V	*E_corr_*, V	*I_corr_*, µA	*OCP*, V	*E_corr_*, V	*I_corr_*, µA
1	No	−1.01	−1.03	44.42	−1.00	−0.98	38.01	−1.11	−1.13	31.63	−1.19	−1.21	29.77
2		−0.99	−1.02	38.31	−0.99	−0.98	17.77	−1.14	−1.12	53.24	−1.21	−1.22	24.42
3		−0.98	−1.02	38.71	−1.01	−1.03	16.03	−1.17	−1.15	44.38	−1.13	−1.17	61.84
1	Yes	−1.00	−1.02	24.12	−1.00	−1.02	17.18	−1.17	−1.16	58.14	−1.15	−1.16	30.38
2		−1.00	−0.99	14.05	−1.02	−1.01	28.85	−1.16	−1.18	55.23	−1.19	−1.14	44.96
3		−1.00	−1.02	28.80	−0.99	−1.00	33.30	−1.16	−1.14	53.84	−1.16	−1.15	39.30

## Data Availability

Data concerning the several measurements are available upon request.
